# Transcutaneous Auricular Vagus Nerve Stimulation for Post-COVID-19 Condition: A Systematic Review and Critical Appraisal of Clinical Evidence

**DOI:** 10.3390/jcm15114247

**Published:** 2026-05-30

**Authors:** Adrian Balan, Giles Graham, Sorin Herban, Marius Marcu, Nini Gheorghe, Gabriela Mara, Florin Claudiu Rasinar, Ana Lascu, Cristian Ion Mot, Traian Flavius Dan, Stefan Mihaicuta, Stefan Marian Frent

**Affiliations:** 1 Center for Research and Innovation in Precision Medicine of Respiratory Diseases, Department of Pulmonology, University of Medicine and Pharmacy Timisoara, 300041 Timisoara, Romania; adrian.balan.umfvbt@gmail.com (A.B.); stefan.mihaicuta@umft.ro (S.M.); frentz.stefan@umft.ro (S.M.F.); 2 Europe to Europe, 78 Providence Park, Southampton SO16 7QW, UK; drgrahamgiles@yahoo.co.uk; 3 Royal Society of Medicine, 1 Wimpole Street, London W1G OAE, UK; 4 Department of Civil Engineering and Installations, Polytechnic University of Timisoara, 300006 Timisoara, Romania; sorin.herban@upt.ro; 5 Department of Computer and Software Engineering, Polytechnic University of Timisoara, 300006 Timisoara, Romania; marius.marcu@cs.upt.ro; 6 Faculty of Medicine, Vasile Goldis Western University of Arad, 310414 Arad, Romania; nini.gheorghe@uvvg.ro (N.G.); mara.gabriela@uvvg.ro (G.M.); 7 Multidisciplinary Doctoral School, Vasile Goldis Western University of Arad, 310414 Arad, Romania; 8 Department of Doctoral Studies, University of Medicine and Pharmacy Timisoara, Eftimie Murgu Square No. 2, 300041 Timisoara, Romania; claudiu.rasinar@umft.ro; 9 Institute for Cardiovascular Diseases of Timisoara, Clinic for Cardiovascular Surgery, 300310 Timisoara, Romania; 10 Department of Functional Sciences, Discipline of Pathophysiology, Centre for Translational Research and Systems Medicine, University of Medicine and Pharmacy Timisoara, Eftimie Murgu Square No. 2, 300041 Timisoara, Romania; 11 Otorhinolaryngology Department, Municipal Emergency Hospital Timisoara, 300041 Timisoara, Romania; ion.mot@umft.ro; 12 Department of Surgery, University of Medicine and Pharmacy Timisoara, Eftimie Murgu Square No. 2, 300041 Timisoara, Romania; 13 Department of Neurosciences-First Division of Neurology, Victor Babes University of Medicine and Pharmacy, 300041 Timisoara, Romania; 14 First Department of Neurology, Pius Branzeu Clinical Emergency County Hospital, 300723 Timisoara, Romania

**Keywords:** post-COVID-19 condition, Long COVID, vagus nerve stimulation, transcutaneous auricular vagus nerve stimulation, taVNS, systematic review, neuromodulation, cholinergic anti-inflammatory pathway, dysautonomia, risk of bias

## Abstract

**Background:** Long COVID, or post-COVID-19 condition (PCC), affects around 36% of individuals following SARS-CoV-2 infection, manifesting as persistent fatigue, cognitive dysfunction, and dysautonomia among its hallmark features. Affecting an estimated 400 million individuals globally, it imposes an annual economic burden exceeding $1 trillion, yet no pharmacological therapy has demonstrated consistent efficacy in adequately powered randomized controlled trials. Transcutaneous auricular vagus nerve stimulation (taVNS) has emerged as a candidate intervention targeting the autonomic dysfunction and neuroinflammation responsible for PCC pathophysiology. **Methods:** We conducted a PRISMA 2020-compliant systematic review (PROSPERO: CRD420261287286) searching PubMed, Scopus, Cochrane, and Web of Science databases from inception to January 2026 for studies evaluating any form of VNS in adults with Long COVID. Risk of bias was assessed using the Cochrane Risk of Bias 2 (RoB 2) tool, the JADAD scale, and the PEDro scale. Certainty of evidence was evaluated using the GRADE framework. Narrative synthesis followed SWiM guidelines. **Results:** Five studies (n = 154 participants) (three randomized controlled trials (RCTs) and two single-arm studies) met inclusion criteria. Three of five studies (60%) were rated high overall risk of bias; only two RCTs achieved “some concerns.” The only adequately double-blinded RCT found no significant between-group differences across all outcomes. Paradoxically, in the best-powered RCT (Percin et al.), sham stimulation produced significantly greater fatigue improvement than active taVNS, despite active taVNS producing significant HRV increases consistent with cardiac autonomic modulation. All efficacy outcomes were rated “very low” certainty (GRADE); safety was rated “low” certainty. **Conclusions:** Currently available evidence supporting the use of taVNS for Long COVID remains limited, and the absence of reliable target engagement markers in the included studies constrains confidence in this approach. Nonetheless, the physiological rationale remains sound, and the favorable safety profile across all included studies supports the feasibility of future investigation. However, given that positive findings were confined to inadequately controlled studies, enthusiasm for further research should be directed first toward mechanistic clarification and rigorous dose-finding work. Large-scale, double-blind, sham-controlled trials incorporating validated markers of vagal engagement are required before taVNS can be firmly recommended for COVID-19 sequelae management.

## 1. Introduction

The coronavirus disease 2019 (COVID-19) pandemic, caused by severe acute respiratory syndrome coronavirus 2 (SARS-CoV-2), has resulted in over 780 million confirmed cases globally [1]. While most individuals recover within weeks, a substantial proportion develop persistent symptoms extending months or years beyond the acute infection, a syndrome termed Long COVID, post-COVID-19 condition (PCC), or post-acute sequelae of SARS-CoV-2 infection (PASC) [2,3]. The most recent pooled prevalence estimate, derived from a meta-analysis of 429 studies encompassing over 2 million confirmed infections, reports a global prevalence of 36% (95% CI, 33–40%) [4]. Population-level analyses estimate a cumulative incidence approaching 400 million affected individuals worldwide, with an annual economic burden exceeding $1 trillion, representing approximately 1% of global GDP [5,6]. In the United States alone, the direct and indirect costs range from $2.0 to $30.8 billion annually, with the vast majority attributable to productivity losses [6]. Long COVID disproportionately affects women, individuals with pre-existing comorbidities, and those who were hospitalized during acute infection [7]. The incidence has declined with Omicron-era infections, and vaccination provides a significant risk reduction; nonetheless, three-year follow-up data confirm persistent disability, with PASC contributing 90.0 disability-adjusted life years per 1000 hospitalized persons annually at three years post-infection [8,9,10].

Long COVID encompasses a heterogeneous constellation of over 200 symptoms affecting virtually every organ system. The most commonly reported manifestations include debilitating fatigue (20–35%), cognitive dysfunction (“brain fog”; approximately 36%), dyspnea (20–24%), post-exertional malaise, sleep disturbance, autonomic dysfunction, and olfactory or gustatory impairment [11,12]. These symptoms significantly impair quality of life, employment, and functional status [13]. The pathophysiology remains incompletely understood, with several interconnected mechanisms identified: persistent viral reservoirs in tissues [14]; immune dysregulation encompassing exhausted T-cell responses, complement activation, and autoantibody formation [15,16]; endothelial dysfunction with fibrin-mediated microclotting and thromboinflammation [17,18,19]; blood–brain barrier disruption with neuroinflammation and glial activation [20]; peripheral serotonin depletion impairing vagal signaling and hippocampal neurogenesis [21]; and mitochondrial dysfunction contributing to fatigue and exercise intolerance [22]. Pharmacovigilance analyses of commonly used COVID-19 treatments, including remdesivir, have confirmed that neuropsychological adverse drug reactions are not attributable to antiviral therapy, reinforcing that the neurological burden of PCC reflects disease-mediated pathophysiology rather than potential treatment toxicity [23]. Of particular mechanistic relevance to the present review, accumulating evidence implicates vagus nerve dysfunction as a central pathophysiological nexus in PCC. Post-mortem studies have detected SARS-CoV-2 RNA with inflammatory cell infiltration in vagus nerves of deceased patients, with inverse correlations between viral load and trans-synaptic signaling genes suggesting dose-dependent axonal dysfunction [24]. Clinical studies have demonstrated vagus nerve thickening on ultrasonography and objective features of vagal impairment in a large cross-sectional PCC cohort (n = 341; 67% exhibiting at least one objective sign of vagal dysfunction) [25], while an integrative model has proposed a self-reinforcing cycle linking chronic vagal dysfunction to hypothalamic–pituitary–adrenal axis impairment and mitochondrial redox failure [26]. Postural orthostatic tachycardia syndrome (POTS) affects approximately 30% of highly symptomatic PCC patients, with broader dysautonomia documented [27,28].

The therapeutic landscape for PCC is characterized by a marked absence of effective pharmacological interventions. Two completed phase 3 randomized controlled trials of nirmatrelvir-ritonavir for established PCC were both negative [29,30]. Metformin demonstrated a 41% reduction in PCC incidence when administered during acute infection, but this applies to prevention, not treatment of established disease [31]. Pulmonary rehabilitation represents the modality with the most replicated evidence [32], while the RECOVER-NEURO trial, evaluating cognitive rehabilitation in 328 participants, found no intervention superior to comparators [33]. Lancet clinical update guidelines explicitly acknowledge the absence of evidence-based pharmacological treatment for PCC [34]. Vagus nerve stimulation (VNS) has been employed therapeutically since 1997 for drug-resistant epilepsy and since 2005 for treatment-resistant depression. The scientific rationale for VNS in inflammatory conditions rests on the cholinergic anti-inflammatory pathway (CAP), initially described by Borovikova and colleagues, who demonstrated that vagal stimulation attenuates the systemic inflammatory response to endotoxin via acetylcholine-mediated inhibition of macrophage cytokine production [35]. The “inflammatory reflex” described by Tracey operates through vagal activation of splenic nerve pathways, culminating in acetylcholine binding to α7 nicotinic acetylcholine receptors on macrophages, suppressing pro-inflammatory cytokine release [36,37]. This pathway was translated to human therapeutics in the recent RESET-RA trial, which demonstrated significant clinical benefit from implanted VNS in rheumatoid arthritis [38]. However, emerging evidence suggests that the classical rodent CAP architecture (requiring a splenic nerve-T-cell relay) may differ substantially in humans [39], and comprehensive meta-analyses have found no consistent evidence for anti-inflammatory effects of non-invasive VNS across human studies [40]. Transcutaneous auricular VNS (taVNS) offers non-invasive access to vagal afferents through the auricular branch of the vagus nerve (ABVN) at the cymba conchae or tragus: structures with documented central projections to the nucleus tractus solitarius [41]. Given the convergence of vagal dysfunction, dysautonomia, and neuroinflammation in PCC pathophysiology, taVNS has been explored as a potential therapeutic modality in several recent pilot studies [42,43,44,45].

No prior PRISMA-compliant systematic review has exclusively evaluated the clinical evidence for VNS in post-COVID-19 condition. A previous narrative overview examined vagal stimulation in Long COVID without formal risk-of-bias assessment or PROSPERO registration [46]. A broader systematic review of non-invasive brain stimulation in PCC included a single taVNS study among 19 studies of heterogeneous modalities and did not apply GRADE evidence profiling [47]. Therefore, this systematic review, the first exclusively examining VNS for PCC, aimed to: (1) identify and critically appraise all available clinical evidence for taVNS as a treatment for Long COVID; (2) assess methodological quality using the RoB 2 tool, JADAD scale, and PEDro scale; (3) evaluate the certainty of evidence using the GRADE framework; (4) synthesize findings across Long COVID symptom domains including fatigue, cognitive dysfunction, autonomic function, olfactory impairment, mood, and sleep quality; (5) evaluate safety and tolerability; and (6) provide evidence-based recommendations for clinical practice and future research.

## 2. Materials and Methods

This systematic review was designed, conducted, and reported in strict accordance with the PRISMA 2020 statement [48] and the Cochrane Handbook for Systematic Reviews of Interventions [49]. The review protocol was prospectively registered in the International Prospective Register of Systematic Reviews (PROSPERO; registration number: CRD420261287286, [50]). The PRISMA 2020 checklist is provided in the Supplementary Materials. All methodological decisions were documented a priori. Post-protocol additions of the JADAD scale, PEDro scale, and GRADE framework are reported transparently in the following sections and justified as enhancements consistent with evolving best practices.

### 2.1. Study Design and Registration

This is a systematic review of interventional studies. The review was prospectively registered in PROSPERO (CRD420261287286, [50]) prior to data extraction. No institutional ethics approval was required, as this study used only previously published aggregate data.

### 2.2. Eligibility Criteria (PICO Framework)

Studies were selected according to the following PICOS criteria, which are summarized in Table 1.

Population: Adults (≥18 years) with Long COVID/PCC, defined as persistent symptoms extending ≥4 weeks beyond confirmed or suspected SARS-CoV-2 infection, consistent with the WHO working definition (ICD-10 U09.9) [51] or equivalent national criteria. Studies were eligible regardless of initial COVID-19 severity. Intervention: Any form of transcutaneous auricular vagus nerve stimulation (taVNS), including percutaneous auricular VNS (paVNS). All stimulation parameters, frequencies, intensities, and treatment durations were eligible. Comparator: Sham stimulation, active control (different stimulation frequency), standard of care, or no comparator. Single-arm studies were included given the nascent evidence base, with appropriate considerations in interpretation. Outcomes: Studies were required to report at least one clinical outcome, including: fatigue (validated scales), cognitive function (validated batteries), mood or anxiety (validated inventories), autonomic function (heart rate variability [HRV] or tilt-table testing), olfactory function (psychophysical testing), sleep quality, quality of life, or safety and tolerability. Study design: Randomized controlled trials, non-randomized controlled studies, and single-arm interventional studies were eligible. Case reports, case series with fewer than five participants, reviews, protocols without results, and preclinical studies were excluded.

### 2.3. Search Strategy

We systematically searched four electronic databases from inception to 20 January 2026 (last search date): PubMed, Scopus, Cochrane CENTRAL, and Web of Science Core Collection. No date restrictions were applied during the search. The full Boolean search strategy for PubMed was: (“COVID-19”[MeSH] OR “COVID-19” OR “SARS-CoV-2”[MeSH] OR “SARS-CoV-2” OR “Coronavirus”[MeSH] OR “Coronavirus” OR “long COVID” OR “post-COVID” OR “PASC” OR “post-acute sequelae”) AND (“Vagus Nerve”[MeSH] OR “Vagus Nerve Stimulation”[MeSH] OR “vagus nerve stimulation” OR “vagal nerve stimulation” OR “transcutaneous auricular vagus” OR “taVNS” OR “auricular vagal”). Adapted strategies using equivalent controlled vocabulary terms were applied in Scopus ((“Vagus Nerve Stimulation” OR “Vagal Stimulation” OR “Transcutaneous Vagus Nerve Stimulation”) AND (“Long COVID” OR “Post-Acute COVID-19 Syndrome” OR “post-COVID”)), Cochrane CENTRAL (“Post-Acute COVID-19 Syndrome” AND “Vagus Nerve Stimulation”, MeSH terms), and Web of Science (ALL = (“long COVID” OR “post-COVID-19” OR “post-acute sequelae” OR “Post-Acute COVID-19 Syndrome”) AND ALL = (“vagus nerve stimulation” OR “vagal nerve stimulation”)). Additional search methods included manual review of reference lists of included studies and relevant reviews (backward citation tracking); forward citation tracking using ResearchRabbit (2025, Version 2025-11-18, last accessed January 2026) and searches of ClinicalTrials.gov, the International Clinical Trials Registry Platform (ICTRP), and the EU Clinical Trials Register for registered, ongoing, or unpublished trials. English language was applied as an eligibility criterion (a potential limiting aspect); the absence of EMBASE from the search strategy due to lack of institutional access is acknowledged as a limitation, though compensatory strategies were systematically applied.

### 2.4. Study Selection and Data Extraction

**Figure 1:**
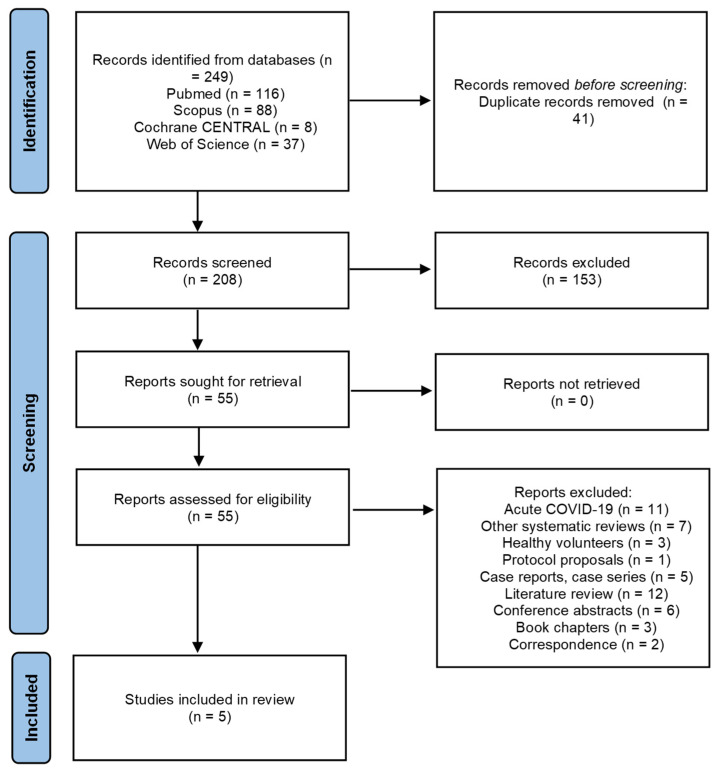
PRISMA 2020 flow diagram.

All records identified from database searches were manually deduplicated. Two reviewers independently screened titles and abstracts against pre-specified eligibility criteria, followed by full-text assessment. Disagreements were resolved through discussion and consensus with the reviewing team, and no major third-party adjudication was required. The study selection process is presented in the PRISMA flow diagram (Figure 1). Data were manually extracted by the authorship team capturing: study identifiers, design characteristics (randomization method, blinding, control type, registration number), participant characteristics (sample size, age, sex, time since acute infection, COVID-19 severity), intervention details (VNS type, device, all stimulation parameters), all reported efficacy outcomes with effect estimates and measures of variability, and safety data. Discrepancies were resolved by consensus referencing to original publications.

### 2.5. Risk of Bias Assessment

Risk of bias was assessed using the Cochrane Risk of Bias 2 (RoB 2) tool for randomized trials [49]. For non-randomized single-arm studies (Zheng et al. and Weise et al. [44,52]), the RoB 2 framework was applied with recognition that Domain 1 (randomization) and Domain 2 (deviations from intended interventions) are rated “high risk” a priori due to the absence of randomization and blinded controls. A novel AI-assisted approach was employed for the initial phase of risk of bias assessment. Each included study was independently analyzed using one AI platform: Claude Sonnet 4.5, Sonnet 4.6, Opus 4.5 and Opus 4.6 (Anthropic, 548 Market Street, San Francisco, CA 94104, USA). Identical verbatim prompts were administered following full-text document upload (“You are a systematic review methodologist trained in the Cochrane Risk of Bias 2 (RoB 2) tool for randomized trials. Assess the uploaded study across all five RoB 2 domains. For each domain, answer the relevant signaling questions, provide a domain-level judgment (Low/Some Concerns/High), and give a concise supporting rationale citing specific text from the paper.”). The resulting assessments were reconciled through two iterative synthesis rounds using Claude Opus 4.6. Also, the RoB2 assessment was independently performed by two of the authors under authorship team supervision before reviewing the AI output. Inter-rater agreement between the two human reviewers was calculated as percentage agreement, given the small number of included studies (n = 5); domain-level agreement and overall judgment agreement was 100%. The two authors who independently performed the RoB 2 assessment critically evaluated AI-generated preliminary outputs against original publications, verified the correct application of signaling questions with reference to the RoB 2 guidance document, and exercised independent judgment on all domain-level verdicts. The inter-rater agreement between AI outcome and human results was calculated for domain-level at 76% and for overall assessment at 80%. Where AI assessments were ambiguous or inconsistent with the source text, human determination was conclusive. The disagreements between AI outputs and human conclusions were discussed within the review team and resolved by majority vote. All final verdicts reflect human judgment and AI results in the preliminary phase were considered only comprehensive drafts. The AI use is disclosed in accordance with emerging transparency standards.

### 2.6. Additional Quality Assessment

The JADAD scale [53,54] and the Physiotherapy Evidence Database (PEDro) scale [55,56] were applied to the three RCTs as supplementary quality instruments. The JADAD scale (range 0–5) evaluates randomization, blinding, and withdrawal reporting. The PEDro scale (range 0–10) evaluates random allocation, concealment, baseline comparability, blinding of subjects/therapists/assessors, follow-up adequacy, intention-to-treat analysis, between-group comparisons, and reporting of point estimates with variability. Both scales were assessed independently by two reviewers; percentage agreement was 100% for both JADAD and PEDro assessments. The two single-arm studies (Zheng et al. and Weise et al. [44,52]) were excluded from JADAD and PEDro assessment as these scales are designed exclusively for randomized designs and their application to non-randomized studies would be methodologically inappropriate. The addition of the JADAD scale, PEDro scale, and GRADE framework (presented in the next paragraph) represents a post-protocol enhancement not pre-specified in the original PROSPERO registration, incorporated to provide a more comprehensive multi-dimensional evaluation of evidence quality, consistent with evolving best practices in systematic review methodology.

### 2.7. Certainty of Evidence (GRADE)

The certainty of evidence was assessed for each pre-specified outcome domain using the Grading of Recommendations, Assessment, Development and Evaluations (GRADE) framework [57,58]. Evidence from RCTs started at “high” certainty and was downgraded based on risk of bias, inconsistency, indirectness, imprecision, and publication bias. Evidence from non-randomized studies started at “low” certainty. GRADE assessments were performed independently by two reviewers with all final determinations reached by consensus under the review team monitoring. Qualitative description of the dual-review consensus process is provided in accordance with GRADE Handbook guidance.

### 2.8. Data Synthesis

Quantitative meta-analytic synthesis was determined to be inappropriate due to substantial clinical heterogeneity (stimulation frequencies ranging from 2 to 80 Hz; treatment durations from a single 10 min session to 12 weeks; diverse outcome domains across studies), methodological heterogeneity (designs ranging from double-blind RCTs to single-arm studies without controls), and outcome heterogeneity (different validated instruments used across the same symptom domain). Data were therefore synthesized narratively, with particular emphasis on the relationship between methodological rigor and reported findings, consistent with Cochrane guidance on Synthesis Without Meta-Analysis (SWiM) [59].

## 3. Results

### 3.1. Study Selection

The systematic database search and supplementary search strategies identified five studies meeting all pre-specified eligibility criteria. The full PRISMA 2020 flow diagram, including record counts at each stage (records identified per database, duplicates removed, records screened, full texts assessed, and reasons for exclusion), is presented in Figure 1.

### 3.2. Study Characteristics

Five studies published between 2022 and 2025 met inclusion criteria, encompassing 154 participants with post-COVID-19 condition [42,43,44,45,52]. The included studies comprised three randomized controlled trials [42,43,45] and two single-arm interventional studies [44,52]. Geographic distribution included the United States (n = 2), Turkey (n = 1), Austria (n = 1), and Germany (n = 1). Sample sizes ranged from 12 to 42 participants (median: 36). Study populations were predominantly female, with three studies enrolling exclusively women. Mean time from acute infection ranged from 7.1 to 20.2 months. Treatment durations varied substantially, from a single 10 min session to 12 weeks of daily stimulation. Study characteristics and stimulation parameters are summarized in Table 2 and Table 3.

### 3.3. Synthesis of Findings by Outcome Domain

#### 3.3.1. Fatigue

Three RCTs examined fatigue as a primary or major secondary outcome, constituting the strongest evidence base within this review. The pattern of findings stratified by methodological quality is diagnostically informative. In Percin et al. [45], despite a priori expectation that active taVNS at therapeutic frequencies (10 Hz) would improve fatigue relative to earlobe sham, the opposite occurred: the sham group demonstrated significantly greater Fatigue Severity Scale (FSS) improvement at treatment completion. This occurred alongside objective evidence of cardiac autonomic modulation in the active group (significant increases in RMSSD (*p* = 0.010) and PNS index (*p* = 0.007) and significant decreases in SNS index (*p* = 0.001) and LF/HF ratio (*p* = 0.002)) while the sham group showed no HRV changes. This fundamental disconnect between successful biomarker response and inferior clinical outcome directly and paradoxically challenges the proposed therapeutic mechanism. In Pfoser-Poschacher et al. [43], modest fatigue improvements (BFI, PCFS) were observed across all three frequency groups (10 Hz, 25 Hz, and 2 Hz control) over 12 weeks, with no significant between-group differences. The equivalence of improvement across all three active-stimulation conditions (including the lowest-frequency “control” arm) could be consistent with non-specific effects (natural recovery, placebo, regression to the mean, study participation) rather than frequency-dependent taVNS efficacy. In Badran et al. [42], the only study with a genuinely credible sham device and true double-blinding, no significant between-group differences in fatigue were identified after 4 weeks. Although underpowered (n = 12), the null finding from this methodologically superior trial aligns with the overall pattern. Collectively, no RCT demonstrated superiority of active taVNS over adequate sham or control stimulation for Long COVID fatigue. A mechanistically distinct rationale for taVNS in fatigue, independent of the CAP, is activation of the ascending reticular activating system (ARAS) via the NTS–locus coeruleus (LC) axis. Vagal afferents projecting to the NTS engage the LC, the primary noradrenergic arousal nucleus, with fMRI studies confirming LC activation following auricular stimulation. This pathway could plausibly reduce fatigue through central arousal modulation rather than peripheral immunomodulation [60].

#### 3.3.2. The Biomarker–Clinical Disconnect

The most consequential finding of this review is the systematic disconnect between HRV-based biomarker changes and clinical outcomes. Percin et al. [45] reported significant HRV improvements following active taVNS, consistent with activation of cardiac parasympathetic pathways. However, these HRV changes did not translate to clinical benefit: the group with no HRV changes (the sham arm) showed superior symptomatic improvement. This paradox is mechanistically important. HRV reflects cardiac parasympathetic modulation through cardiac branches, not splenic efferent activation: the pathway through which the CAP suppresses pro-inflammatory cytokine production [61,62]. The auricular branch of the vagus nerve is exclusively afferent, projecting centrally to the nucleus tractus solitarius [41]; whether this afferent input is sufficient to engage the efferent splenic arc of the inflammatory reflex in humans remains unresolved. Emerging evidence further complicates the classical model: recent work suggests that in humans, the splenic CAP may operate through noradrenergic signaling directly onto myeloid cells rather than the T-cell relay described in rodents [39]. Furthermore, comprehensive meta-analytic evidence has found no consistent anti-inflammatory effects of non-invasive VNS across human studies [40]. Together, these observations suggest that HRV increases cannot serve as a validated surrogate for clinically relevant vagal anti-inflammatory engagement, and that the mechanistic rationale for taVNS in PCC rests on a translational assumption that current clinical evidence provides limited support.

#### 3.3.3. Cognitive Function

Zheng et al. [52] reported statistically significant improvements across multiple NIH Cognitive Toolbox domains in their single-arm study of 24 female participants with Long COVID. However, without a concurrent control group, these improvements cannot be distinguished from practice effects on repeated cognitive testing, natural recovery, placebo effects, or regression to the mean. In contrast, Badran et al. [42] included cognitive assessments as secondary outcomes within their double-blind RCT and found no significant between-group differences. The contrast between positive findings in the uncontrolled study and null findings in the controlled trial closely follows the pattern of how non-specific effects produce apparent efficacy signals in uncontrolled designs.

#### 3.3.4. Olfactory Function

Weise et al. [44] examined acute taVNS effects on olfactory function in 40 participants with post-COVID anosmia or hyposmia using Sniffin’ Sticks TDI scoring before and immediately after a single 10 min stimulation. The study is notable for targeting a distinct PCC domain and employing psychophysical rather than exclusively self-reported outcome measures. However, without a sham control, randomization, or longitudinal follow-up, the design cannot differentiate stimulation-specific effects from test–retest improvement, expectation effects, natural variation across sessions, or assessor influence. A single-session before–after design represents a relatively limited evidence architecture for establishing treatment efficacy.

#### 3.3.5. Mood, Sleep, and Quality of Life

Badran et al. [42] measured anxiety (GAD-7) and depression (PHQ-9) as co-primary outcomes in their double-blind RCT and found no significant between-group differences after 4 weeks. Zheng et al. [52] reported improvements in anxiety, depression, and sleep quality in their uncontrolled pilot, but without a comparator these are uninterpretable as treatment effects. Pfoser-Poschacher et al. [43] found improvements in Insomnia Severity Index scores across all three frequency conditions without significant between-group differences, again consistent with non-specific or time effects rather than frequency-specific taVNS efficacy.

#### 3.3.6. Safety and Tolerability

All five studies consistently reported that taVNS was well-tolerated with no serious adverse events among 154 participants. Badran et al. [42] reported two instances of mild skin irritation at the stimulation site, with no unanticipated adverse events and no cardiac adverse events confirmed across continuous heart rate monitoring throughout the trial. Percin et al. [45] reported that no subjects experienced side effects with either active or sham taVNS. Pfoser-Poschacher et al. [43] reported no serious adverse events or suspected unexpected serious adverse reactions; non-serious effects noted by participants included ear pain, tingling, pulsating sensations, itching, and sleep disturbances, none of which led to discontinuation. No bradycardia or concerning cardiovascular effects were reported in any study. This safety profile is consistent with the broader taVNS literature, in which a systematic review and meta-analysis encompassing 177 studies and over 6000 participants found no significant difference in adverse event risk between active taVNS and sham and no confirmed causal serious adverse events [63,64]. Small sample sizes and short follow-up durations preclude detection of rare or long-term effects, and prospective safety monitoring in future trials remains warranted.

### 3.4. Risk of Bias Assessment

Risk of bias assessments are summarized in Table 4, with detailed domain-level judgments and supporting rationales in Table 5. Overall, three of five studies (60%) received an overall “high” risk of bias rating. Two studies received “some concerns.” No study achieved “low” risk of bias across all five RoB 2 domains. The predominant sources of bias were inadequate blinding procedures (Domain 2), reliance on subjective outcome measures in inadequately blinded studies (Domain 4), and the absence of pre-specified statistical analysis plans with appropriate multiplicity correction (Domain 5). The primary disagreement between the AI results and human conclusion was around the D4 and D5 domains. The AI’s output exhibited notable inaccuracies, including misreading and omission of information from the raw datasets, as well as misunderstandings of some domain-specific concepts. Additionally, the AI demonstrated an incorrect interpretation and application of the analytical algorithm, leading to methodological inconsistencies. These discrepancies highlight the need for careful human oversight when integrating AI-assisted analysis into specialized evaluative processes.

### 3.5. JADAD Quality Assessment

JADAD scale results for the three RCTs are presented in Table 6. Scores ranged from 2 to 5. Only Badran et al. [42] achieved the maximum score of 5, reflecting fully described and methodologically appropriate randomization, genuine double-blinding with a credible sham device, and complete reporting of withdrawals. Percin et al. [45] scored 3, losing points for the absence of double-blinding (described explicitly as single-blind). Pfoser-Poschacher et al. [43] scored 2; the study claimed blinding but received a deduction for an inappropriate blinding method (2 Hz active electrical stimulation as control rather than true sham), which we considered to not constitute genuine blinding, as it may exert unknown vagal effects, which is also acknowledged as a limitation by the investigators. The two single-arm studies were excluded from JADAD assessment as this scale is designed exclusively for randomized controlled trials.

### 3.6. PEDro Quality Assessment

PEDro scores for the three RCTs are presented in Table 7. Scores ranged from 6 to 10 (median: 8). Badran et al. [42] achieved the maximum score of 10/10, reflecting rigorous methodology across all PEDro criteria. Percin et al. [45] scored 8/10, with deductions for absent allocation concealment and absent therapist blinding. Pfoser-Poschacher et al. [43] scored 6/10, with an additional deduction for unclear assessor blinding. These scores corroborate the RoB 2 and JADAD assessments, confirming Badran et al. [42] as the methodologically strongest study in this evidence base, and the only study showing null results.

### 3.7. GRADE Evidence Profile

The GRADE assessment for each pre-specified outcome domain is presented in Table 8. All efficacy outcomes were rated “very low” certainty, and safety was rated “low” certainty. Consistent downgrading reflected: pervasive high or unclear risk of bias across the evidence base; inconsistency between controlled and uncontrolled study findings; small sample sizes producing wide uncertainty; and near-universal reliance on subjective, self-reported outcome measures highly susceptible to placebo effects in inadequately blinded studies. Also, in Table 9 the GRADE Summary of Findings report can be found.

## 4. Discussion

In this systematic review (the first PRISMA-compliant review exclusively evaluating VNS for post-COVID-19 condition), we identified five studies encompassing 154 participants. The central finding is that three of five studies (60%) exhibited high overall risk of bias, positive findings were confined exclusively to inadequately blinded or uncontrolled studies, and the two studies with adequate or near-adequate blinding showed null or paradoxically negative results. This pattern is more suggestive of bias influenced efficacy signals than of a bona fide therapeutic effect, indicating a need for confirmatory studies.

### 4.1. Principal Findings and the Pathophysiological Challenge

Two critical observations fundamentally challenge the rationale for taVNS in PCC. First, the Percin et al. trial [45] demonstrated HRV increases consistent with cardiac parasympathetic modulation yet paradoxically superior clinical outcomes in the sham group with no HRV changes. This creates a logical paradox: if taVNS activates vagal pathways but this activation is associated with inferior clinical outcomes compared to sham, the therapeutic hypothesis could be contradicted rather than supported. This paradoxical finding warrants interpretive restraint before it is interpreted as definitive evidence against taVNS as a therapeutic modality. TaVNS may successfully activate auricular vagal afferents, as the HRV data suggest, while the pathophysiology of Long COVID fatigue in this cohort does not primarily implicate CAP dysregulation given that post-COVID fatigue is sustained by heterogeneous mechanisms. Even if the CAP is a valid target, the stimulation protocols evaluated to date may be insufficient to translate physiological engagement into clinical benefit, as neuromodulatory interventions frequently exhibit duration-response relationships. Second, Pfoser-Poschacher et al. [43] found equivalent improvements across all three stimulation frequencies, including their lowest-frequency “control” condition, inconsistent with a dose- or frequency-dependent mechanism and more consistent with non-specific effects. These observations suggest caution in interpreting taVNS efficacy for PCC and indicate that the hypothesis (that auricular vagal stimulation produces clinically relevant modulation of the inflammatory reflex) merits additional, rigorously controlled study. The afferent-only ABVN pathway [41] must traverse multiple synaptic relays before potentially engaging efferent immunomodulatory circuits, and whether sufficient signal propagation occurs to activate the splenic CAP in humans with PCC remains a critically unresolved question. Parallel findings from the acute COVID-19 VNS literature reinforce this concern: Corrêa et al. [65] reported unchanged HRV despite claimed anti-inflammatory effects, suggesting either non-vagal mediation or systemic inconsistency in the relationship between auricular stimulation and the inflammatory pathway. The current evidence therefore challenges specific assumptions embedded in the therapeutic hypothesis without negating the broader rationale for vagal neuromodulation in post-COVID condition.

### 4.2. The Blinding Problem

Adequate blinding emerges as the critical methodological determinant separating positive from null findings in this evidence base. We consider that only Badran et al. [42] achieved genuine double-blinding through a credible sham device engineered to deliver matched sensory characteristics. Pfoser-Poschacher et al. [43] used 2 Hz active electrical stimulation as their “control,” meaning all groups received perceptible stimulation, precluding any blinded comparison. Zheng et al. [52] and Weise et al. [44] lacked control groups entirely. This blinding deficiency is particularly consequential given that all five studies relied substantially on subjective, self-reported outcome measures (fatigue scales, mood questionnaires, cognitive battery performance) that are known to be among the most responsive to placebo effects and expectation bias [66]. The credibility of a sham control is itself a critical methodological parameter: sham stimulation that participants can distinguish from active treatment is effectively an unblinded comparison. Although designing a credible sham is challenging (active taVNS produces obligatory sensory phenomena that cannot be eliminated without reducing stimulation to sub-perceptual intensities), future trials should adopt sham protocols that match the sensory profile of active stimulation, including matched electrode impedance and sub-threshold current delivery, and must incorporate a formal blinding integrity assessment, reporting the proportion of participants correctly and identifying their allocation.

### 4.3. Stimulation Parameter Heterogeneity and the Dose-Finding Gap

Substantial heterogeneity existed across stimulation protocols without mechanistic rationale for parameter selection. Frequencies ranged 40-fold (2–80 Hz); pulse widths varied three-fold (180–500 μs); session durations ranged from 10 min to 120 min; and total treatment durations ranged from a single session to 84 days of daily stimulation. The 80 Hz stimulation employed by Weise et al. [44] is notably discordant with the 10–25 Hz range used in other studies and with established optimal frequency parameters for VNS in other indications. No dose-finding or dose-escalation study specific to PCC has been conducted. Parameter selection across these trials appears largely extrapolated from other conditions (epilepsy, depression, acute COVID-19) rather than derived from PCC-specific mechanistic studies. This heterogeneity both complicates evidence synthesis and signals the premature stage of clinical development.

### 4.4. Comparison with the Acute COVID-19 VNS Literature

The pattern identified in this review closely parallels findings from evaluations of VNS for acute COVID-19. A systematic review of VNS in acute COVID-19 [67] identified six RCTs with 83% exhibiting high risk of bias; positive findings were confined to methodologically weaker, inadequately blinded studies, while the single adequately double-blinded trial (Rangon et al., SOS COVID-19) [68] found null results across all primary outcomes. A meta-analysis of four RCTs examining inflammatory markers [69] found a significant increase in IL-10 but no significant changes in CRP, IL-6, or D-dimer: a highly selective biomarker pattern that does not support clinically meaningful anti-inflammatory engagement. This consistent pattern across both acute and post-acute COVID-19 VNS applications suggests systematic methodological issues that are not indication-specific. This convergence argues for the pattern to be interpreted as reflecting the general susceptibility of poorly blinded, subjective-outcome trials to reporting artificially positive results.

### 4.5. Strengths and Limitations

The principal strengths of this review include: its status as the first PRISMA 2020-compliant systematic review exclusively examining VNS for post-COVID-19 condition; the application of three independent quality assessment instruments (RoB 2, JADAD, PEDro) alongside GRADE evidence profiling, providing a multi-dimensional quality characterization; the novel application of a multi-AI tool initial screening approach with full human validation, with transparent methodology disclosure; prospective PROSPERO registration; comprehensive supplementary searching of trial registries; and citation tracking to minimize publication bias. The review was conducted following the pre-specified protocol, without the need for protocol deviations or amendments with transparent reporting of all post-protocol methodological enhancements.

Limitations include the small number of eligible studies (n = 5), substantial clinical and methodological heterogeneity that precluded quantitative synthesis, and sample sizes ranging from 12 to 42 participants: all underpowered for reliable efficacy estimation. The exclusion of EMBASE from the database search due to the absence of institutional access is acknowledged as a residual limitation, though compensatory strategies including registry searches and forward and backward citation tracking were systematically employed. Restriction to English-language publications may have excluded relevant studies from non-English-language settings. Publication bias cannot be excluded, though registered trial searches identified ongoing studies consistent with an active but incomplete literature. All five included studies were published between 2022 and 2025, reflecting a rapidly evolving field where evidence accrues continuously.

### 4.6. Recommendations for Future Research

Before embarking on large definitive efficacy trials, several prior questions require resolution. Mechanistic studies should investigate: whether the splenic efferent arc of the inflammatory reflex is actually dysregulated and activatable in PCC; whether auricular stimulation (mediated exclusively through afferent ABVN projections) can engage this efferent pathway at sufficient magnitude in humans; and whether such engagement, if achievable, translates to symptom improvement in the specific context of PCC pathophysiology. Dose-finding studies are urgently needed to establish optimal frequency, pulse width, intensity, and duration parameters for PCC specifically. Essential design features for definitive trials include: adequate sample size based on conservative effect size estimates from genuinely controlled (not uncontrolled) studies; genuine double-blinding with a sham device engineered to match the sensory characteristics of active stimulation and with formal blinding success assessment; objective or observer-rated outcome measures alongside patient-reported instruments; pre-specified primary outcomes with appropriate correction for multiplicity; longitudinal follow-up of at least 3–6 months; inclusion of mechanistic biomarkers to confirm target engagement; and independent funding sources to minimize conflicts of interest.

## 5. Conclusions

This systematic review of five studies (n = 154 participants) evaluating transcutaneous auricular vagus nerve stimulation for post-COVID-19 condition found insufficient evidence to support taVNS as an effective treatment. Three of five studies (60%) were rated high risk of bias by the Cochrane RoB 2 framework, and the two adequately controlled trials showed either null effects or paradoxically superior outcomes with sham treatment. The biomarker–clinical disconnect observed in the best-powered trial (where HRV changes consistent with cardiac autonomic modulation did not translate to clinical benefit, and the sham group showed significantly greater fatigue improvement) represents a fundamental challenge to the proposed therapeutic rationale. JADAD and PEDro scores confirmed significant quality disparities across the RCTs, and GRADE assessment rated all efficacy outcomes as “very low” certainty. This pattern parallels findings from systematic evaluations of VNS in acute COVID-19, suggesting methodological issues that are systematic across the VNS–COVID literature rather than indication-specific. While the pathophysiological rationale targeting autonomic dysfunction and neuroinflammation in PCC remains scientifically plausible, and the safety profile appears favorable across all included studies, rigorously designed double-blind RCTs with objective outcome measures and credible sham controls are essential before any clinical recommendation can be made.

## Figures and Tables

**Table 1:** PICO framework for study inclusion.

Parameter	Definition
**Population (P)**	Adults (≥18 years) with Long COVID/post-COVID-19 condition (PCC; ICD-10 U09.9); persistent symptoms ≥4 weeks post-infection; all initial severity levels.
**Intervention (I)**	Any form of transcutaneous auricular vagus nerve stimulation (taVNS), including percutaneous auricular VNS; all stimulation parameters eligible.
**Comparator (C)**	Sham stimulation, active control, standard of care, or no comparator (single-arm studies eligible).
**Outcomes (O)**	Fatigue, cognitive function, mood/anxiety, autonomic function (HRV), olfactory function, sleep quality, quality of life, safety/tolerability.
**Study design (S)**	Randomized controlled trials; non-randomized controlled studies; single-arm interventional studies (≥5 participants).

**Table 2:** Characteristics of included studies.

Study	Design	N	Intervention	Duration	Primary Outcomes	Registration
Badran 2022 [42]	Double-blind RCT	12	taVNS 25 Hz, 500 μs, 60 min 2×/day (cymba conchae); sham: matched device, sub-threshold	4 weeks (28 days)	GAD-7, PHQ-9, FSS, fatigue NRS	NCT04638673
Percin 2025 [45]	Single-blind RCT	42	taVNS 10 Hz, 300 μs, 0.5–12 mA, 30 min/day; sham: no active stimulation	20 days	FSS, HRV (RMSSD, SDNN, LF/HF)	NCT05679505
Pfoser-Poschacher 2025 [43]	RCT (3-arm, pilot)	36	taVNS 10, 25, or 2 Hz, ~9.5 mA, 30 min/day (TENSeco device); 2 Hz arm = active control	12 weeks (84 days)	BFI, PCFS, HRV, ISI	NCT05918965
Zheng 2024 [52]	Single-arm pilot	24	taVNS 25 Hz, 250 μs, 13.64 mA, 30 min 2×/day (Parasym AVNT; cymba conchae)	10 days	NIH Cognitive Toolbox, PROMIS	NCT05225220
Weise 2025 [44]	Single-session uncontrolled	40	taVNS 80 Hz, 180 μs, 10–20 mA, 10 min (TENS ECO-2)	Single session	Sniffin’ Sticks (TDI score)	None

BFI, Brief Fatigue Inventory; FSS, Fatigue Severity Scale; GAD-7, Generalized Anxiety Disorder-7; HRV, heart rate variability; ISI, Insomnia Severity Index; NRS, Numeric Rating Scale; PCFS, Post-COVID-19 Functional Status; PHQ-9, Patient Health Questionnaire-9; PROMIS, Patient-Reported Outcomes Measurement Information System; RCT, randomized controlled trial; taVNS, transcutaneous auricular vagus nerve stimulation; TDI, Threshold-Discrimination-Identification.

**Table 3:** Stimulation parameters across included studies.

Study	Frequency (Hz)	Pulse Width (μs)	Intensity (mA)	Session Length	Total Duration	Device
Badran 2022 [42]	25	500	2× sensory threshold	60 min (2×/day)	28 days	Soterix Medical
Percin 2025 [45]	10	300	0.5–12	30 min/day	20 days	Vagustim
Pfoser-Poschacher 2025 [43]	10, 25, or 2	Not specified	~9.5	30 min/day	84 days	TENSeco
Zheng 2024 [52]	25	250	13.64 (mean)	30 min (2×/day)	10 days	Parasym AVNT
Weise 2025 [44]	80	180	10–20	10 min	Single session	TENS ECO-2

**Table 4:** Risk of bias summary by RoB 2 domain.

Study	D1: Randomization	D2: Deviations	D3: Missing Data	D4: Measurement	D5: Reporting	Overall
Badran 2022 [42]	Low	Low	Low	Some concerns	Low	Some concerns
Percin 2025 [45]	Some concerns	Some concerns	Low	Low	Low	Some concerns
Pfoser-Poschacher 2025 [43]	Some concerns	High	Low	Some concerns	Low	High
Zheng 2024 [52] *	High	High	Some concerns	High	Low	High
Weise 2025 [44] *	High	High	Low	Some concerns	High	High

* Single-arm studies classified “high risk” a priori in Domains 1 and 2 owing to absence of randomization and controls. D1–D5: RoB 2 domains 1–5. Overall judgment: Low = low risk across all domains; some concerns = at least one domain with some concerns, none high; high = at least one domain with high risk.

**Table 5:** Detailed RoB 2 domain judgments with supporting rationales.

Study	Domain: Judgment	Supporting Rationale
Badran 2022 [42]	D1: Low	Coded allocation preventing investigators unblinding; no baseline imbalances detected. Allocation concealment not fully described.
	D2: Low	Coded allocation system precluded foreknowledge by both staff and participants: group-specific stimulation codes were issued only after perceptual threshold establishment, and neither enrolling personnel nor participants were informed which code set (A or B) delivered active versus sham stimulation. No baseline imbalances detected.
	D3: Low	Complete follow-up (12/12 participants retained). No missing outcome data.
	D4: Some Concerns	Primary outcomes (GAD-7, PHQ-9, fatigue scales) remain self-reported and susceptible to residual placebo effects even with adequate blinding.
	D5: Low	Prospectively registered (NCT04638673). Framed appropriately as a feasibility/pilot study.
Percin 2025 [45]	D1: Some Concerns	Randomization described; groups similar at baseline. Allocation concealment not fully detailed.
	D2: Some Concerns	Described as “single-blind.”
	D3: Low	No dropouts among 42 participants. Complete data for all outcomes.
	D4: Low	Appropriate measuring method, no difference between groups, and assessor blinded trial.
	D5: Low	Prospectively registered (NCT05679505). Multiple outcomes reported without pre-specified multiplicity correction. Post hoc power calculations acknowledge smaller-than-anticipated effects.
Pfoser-Poschacher 2025 [43]	D1: Some Concerns	Randomization mentioned but not described. Allocation concealment incompletely reported.
	D2: High	2 Hz stimulation used as “control” constitutes active electrical stimulation, not true sham, as it might cause unknown effects even engaging vagal pathways. All groups received perceptible sensory stimulation. Participants could plausibly differentiate frequency conditions, producing performance bias.
	D3: Low	35/36 participants (97.2%) completed follow-up. Minimal attrition.
	D4: Some Concerns	Subjective fatigue measures (BFI, PCFS) in an inadequately blinded study. All frequency groups improved similarly, consistent with non-specific or placebo effects rather than frequency-specific taVNS efficacy. No information about assessor blinding.
	D5: Low	Registered (NCT05918965). Multiple outcomes analyzed without pre-specified analysis plan.
Zheng 2024 [52]	D1: High	Single-arm design without randomization or control group. Confounding cannot be excluded.
	D2: High	No control group. Cannot distinguish treatment-specific effects from natural recovery, practice effects, placebo, regression to the mean, or Hawthorne effects.
	D3: Some Concerns	Variable sample sizes across outcomes (n = 18–24).
	D4: High	Cognitive assessments performed without blinding or control group. Practice effects from repeated cognitive testing cannot be excluded.
	D5: Low	Registered (NCT05225220). Multiple cognitive, mood, and sleep outcomes analyzed.
Weise 2025 [44]	D1: High	Single-session uncontrolled design without randomization or comparator.
	D2: High	No control group. Single-session before–after design cannot distinguish specific stimulation effects from test–retest effects, expectation, natural variation, or assessor prompting.
	D3: Low	Complete data for all 40 participants.
	D4: Some Concerns	Psychophysical olfactory testing (Sniffin’ Sticks) is more objective than questionnaires but still involves subjective detection responses and was unblinded.
	D5: High	No trial registration. Multiple olfactory and attentional outcomes analyzed without pre-specification.

**Table 6:** JADAD quality assessment of randomized controlled trials.

Study	Randomization Described (+1)	Randomization Appropriate (+1/−1)	Double-Blind Described (+1)	Blinding Appropriate (+1/−1)	Withdrawals (+1)	Total (/5)
Badran 2022 [42]	+1	+1	+1	+1	+1	5
Percin 2025 [45]	+1	+1	0 (single-blind)	0	+1	3
Pfoser-Poschacher 2025 [43]	+1	0	+1 (claimed)	−1 (2 Hz active ≠ sham)	+1	2

Zheng et al. and Weise et al. [44,52] excluded from JADAD assessment (non-randomized designs).

**Table 7:** PEDro quality assessment of randomized controlled trials.

Criterion	Badran 2022 [42]	Percin 2025 [45]	Pfoser-Poschacher 2025 [43]
Random allocation	Yes	Yes	Yes
Concealed allocation	Yes (coded)	No (unclear)	No (unclear)
Baseline similarity	Yes	Yes	Yes
Subject blinding	Yes (sham device)	Yes	No (active control)
Therapist blinding	Yes (automated)	No	No
Assessor blinding	Yes	Yes (assessor-blind)	No (unclear)
>85% follow-up	Yes (100%)	Yes (100%)	Yes (97%)
Intention-to-treat	Yes	Yes	Yes
Between-group comparison	Yes	Yes	Yes
Point estimates + variability	Yes	Yes	Yes
Total Score (/10)	10	8	6

Criterion 1 (eligibility criteria specified) is not counted toward the PEDro total score. Zheng et al. and Weise et al. [44,52] excluded from PEDro assessment (non-randomized designs).

**Table 8:** GRADE evidence profile.

Outcome	Studies (N)	Risk of Bias	Inconsistency	Indirectness	Imprecision	Publication Bias	Starting Level	Certainty
**Fatigue**	3 RCTs (n = 90)	Serious (−2)	Serious (−1)	Not serious	Serious (−1)	Undetected	High	VERY LOW ⊕◯◯◯
**Cognitive function**	1 RCT + 1 SA (n = 36)	Serious (−1)	Serious (−1)	Not serious	Serious (−1)	Undetected	Low †	VERY LOW ⊕◯◯◯
**HRV/Autonomic**	2 RCTs (n = 78)	Serious (−1)	Not serious	Serious (−1) ‡	Serious (−1)	Undetected	High	VERY LOW ⊕◯◯◯
**Mood/Anxiety**	1 RCT + 1 SA (n = 36)	Serious (−1)	Serious (−1)	Not serious	Serious (−1)	Undetected	Low †	VERY LOW ⊕◯◯◯
**Olfactory function**	1 SA (n = 40)	Very serious (−2)	N/A	Not serious	Serious (−1)	Undetected	Very Low †	VERY LOW ⊕◯◯◯
**Safety**	5 studies (n = 154)	Not serious	Not serious	Not serious	Serious (−1)	Undetected	Low †	LOW ⊕⊕◯◯

† Starting certainty “low” due to inclusion of non-randomized (single-arm) study evidence. ‡ Indirectness downgraded for HRV: biomarker changes did not correlate with clinical outcomes; HRV reflects cardiac parasympathetic modulation although it is not a validated surrogate for immunologically relevant vagal engagement [61]. SA, single-arm study; RCT, randomized controlled trial. Certainty symbols: ⊕⊕⊕⊕ high; ⊕⊕⊕◯ moderate; ⊕⊕◯◯ low; ⊕◯◯◯ very low.

**Table 9:** GRADE summary of findings—taVNS for post-COVID-19 condition evidence profile.

Outcome (Follow-Up)	Participants (Studies)	Outcome Measures	Direction of Effect	Certainty (GRADE)	Basis for Rating
**Fatigue ≤12 weeks**	n = 90 3 RCTs ^a^	FSS BFI PCFS	No RCT demonstrated superiority of active taVNS. In Percin et al., sham outperformed active taVNS on FSS despite HRV changes consistent with autonomic modulation (RMSSD↑, PNS index↑). Pfoser-Poschacher et al. found equivalent improvements across all three frequency arms including the 2 Hz control. Badran et al. (double-blind) found no between-group difference. Active taVNS was not superior to sham.	VERY LOW ⊕◯◯◯	−2 Risk of bias: inadequate blinding; subjective outcomes −1 Inconsistency: paradoxical sham superiority (Percin) vs. equivalent within-group gains (Pfoser-Poschacher) −1 Imprecision: n = 12–42 per study
**Cognitive Function 10 days–4 weeks**	n = 36 1 RCT + 1 SA ^b^	NIH Toolbox GAD-7/PHQ-9 MOCA	Zheng et al. (single-arm, n = 24) found significant within-group gains in fluid cognition and processing speed sustained at 1-month follow-up. Badran et al. (double-blind RCT, n = 12) found no between-group difference. Within-group improvements are uninterpretable without a credible comparator. No controlled evidence of benefit.	VERY LOW ⊕◯◯◯	Starting level: Low (SA study included) −1 Risk of bias: uncontrolled primary study −1 Inconsistency: SA gains not replicated in RCT −1 Imprecision: n = 12–24
**HRV/Autonomic Function 20 days–12 weeks**	n = 78 2 RCTs ^c^	RMSSD LF/HF ratio PNS/SNS indices	Active taVNS significantly improved HRV parameters vs. sham in Percin et al. consistent with autonomic modulation. HRV was stable across all arms in Pfoser-Poschacher et al. Critically, HRV improvement in Percin was not accompanied by clinical benefit: sham produced superior fatigue outcomes. HRV indexes cardiac parasympathetic tone, not the splenic efferent pathway underlying the cholinergic anti-inflammatory mechanism.	VERY LOW ⊕◯◯◯	−1 Risk of bias: single-blind design (Percin) −1 Indirectness: HRV not a validated surrogate for immunologically relevant vagal engagement; biomarker–clinical dissociation −1 Imprecision: n = 36–42
**Mood/Anxiety 10 days–4 weeks**	n = 36 1 RCT + 1 SA ^b^	GAD-7 PHQ-9 PROMIS Anxiety & Depression	Zheng et al. (single-arm) reported significant within-group improvements in PROMIS anxiety and depression. Badran et al. (double-blind RCT) found no between-group difference in GAD-7 or PHQ-9. No controlled evidence of benefit.	VERY LOW ⊕◯◯◯	Starting level: Low (SA study included) −1 Risk of bias: no blinding in primary study −1 Inconsistency: SA gains absent in RCT −1 Imprecision: n = 12–24
**Olfactory Function Single session**	n = 40 1 SA ^d^	Sniffin’ Sticks TDI (Discrimination, Threshold, Identification)	Weise et al. (single-session uncontrolled, n = 40) found improved olfactory discrimination in PCC patients vs. no change in healthy. Threshold and identification did not improve. Absence of sham control and single-session design preclude causal attribution. Preliminary signal only.	VERY LOW ⊕◯◯◯	Starting level: Very Low (uncontrolled single session) −2 Risk of bias: no sham; practice effects uncontrolled −1 Imprecision: single time point; effect isolated to one subdomain
**Safety and Tolerability 10 days–12 weeks**	n = 154 5 studies ^e^	Adverse event reporting (unsystematic)	No serious adverse events in any study. Mild, transient effects in a minority of participants: ear discomfort, skin tingling, headache, insomnia (rates 27–42% mild-only in Pfoser-Poschacher; 2 skin-irritation events in Badran). No bradycardic events in any monitored study. Well-tolerated at stimulation parameters used.	LOW ⊕⊕◯◯	Starting level: Low (non-randomized studies included; AE reporting unsystematic) −1 Imprecision: n = 154 insufficient for rare AE detection; follow-up ≤12 weeks

GRADE certainty ratings: HIGH ⊕⊕⊕⊕; MODERATE ⊕⊕⊕◯; LOW ⊕⊕◯◯; VERY LOW ⊕◯◯◯. Very Low = any estimate of effect is very uncertain; Low = further research very likely to have an important impact. ^a^ Badran 2022 [42] (n = 12); Percin 2025 [45] (n = 42); Pfoser-Poschacher 2025 [43] (n = 36). ^b^ Badran 2022 [42] (RCT, n = 12); Zheng 2024 [52] (single-arm, n = 24). ^c^ Percin 2025 [45] (n = 42); Pfoser-Poschacher 2025 [43] (n = 36). ^d^ Weise 2025 [44] (n = 40). ^e^ All five included studies. AE, adverse event; BFI, Brief Fatigue Inventory; FSS, Fatigue Severity Scale; GAD-7, Generalized Anxiety Disorder-7; HRV, heart rate variability; LF/HF, low-/high-frequency ratio; MOCA, Montreal Cognitive Assessment; PCFS, Post-COVID-19 Functional Status; PHQ-9, Patient Health Questionnaire-9; PNS, parasympathetic nervous system; PROMIS, Patient-Reported Outcomes Measurement Information System; RCT, randomized controlled trial; RMSSD, root mean square of successive differences; SA, single-arm study; SNS, sympathetic nervous system; taVNS, transcutaneous auricular vagus nerve stimulation; TDI, Threshold-Discrimination-Identification.

## Data Availability

All data generated or analyzed during this study are included in this published article and its Supplementary Materials. The PROSPERO registration for this review is publicly accessible at https://www.crd.york.ac.uk/prospero/display_record.php?ID=CRD420261287286 (Accessed on 20 April 2026).

## Supplementary Materials

The following supporting information can be downloaded at: https://www.mdpi.com/article/10.3390/jcm15114247/s1, PRISMA 2020 Checklist—Transcutaneous Auricular Vagus Nerve Stimulation for Post-COVID-19 Condition: A Systematic Review and Critical Appraisal of Clinical Evidence.

## Abbreviations

The following abbreviations are used in this manuscript:

ABVN	Auricular branch of the vagus nerve
ARAS	Ascending reticular activating system
BFI	Brief Fatigue Inventory
CAP	Cholinergic anti-inflammatory pathway
CENTRAL	Cochrane Central Register of Controlled Trials
CI	Confidence interval
FSS	Fatigue Severity Scale
GAD-7	Generalized Anxiety Disorder-7 scale
GRADE	Grading of Recommendations, Assessment, Development and Evaluations
HRV	Heart rate variability
JADAD	Jadad quality assessment scale
LC	Locus coeruleus
LF/HF	Low-frequency/high-frequency (HRV ratio)
NTS	Nucleus tractus solitarius
PASC	Post-acute sequelae of SARS-CoV-2
PCC	Post-COVID-19 condition
PCFS	Post-COVID-19 Functional Status scale
PEDro	Physiotherapy Evidence Database scale
PHQ-9	Patient Health Questionnaire-9
PRISMA	Preferred Reporting Items for Systematic Reviews and Meta-Analyses
PROSPERO	International Prospective Register of Systematic Reviews
RCT	Randomized controlled trial
RMSSD	Root mean square of successive differences (HRV parameter)
RoB 2	Cochrane Risk of Bias 2 tool
SARS-CoV-2	Severe acute respiratory syndrome coronavirus 2
SDNN	Standard deviation of normal-to-normal intervals (HRV parameter)
SWiM	Synthesis Without Meta-Analysis
taVNS	Transcutaneous auricular vagus nerve stimulation
VNS	Vagus nerve stimulation
